# *In Silico* target fishing: addressing a “Big Data” problem by ligand-based similarity rankings with data fusion

**DOI:** 10.1186/1758-2946-6-33

**Published:** 2014-06-18

**Authors:** Xian Liu, Yuan Xu, Shanshan Li, Yulan Wang, Jianlong Peng, Cheng Luo, Xiaomin Luo, Mingyue Zheng, Kaixian Chen, Hualiang Jiang

**Affiliations:** 1 Drug Discovery and Design Center, State Key Laboratory of Drug Research, Shanghai Institute of Materia Medica, Chinese Academy of Sciences, 555 Zuchongzhi Road, Shanghai 201203, China; 2 School of Life Science and Technology, ShanghaiTech University, Shanghai 200031, China

**Keywords:** Target fishing, Big data, Molecular fingerprints, Data fusion, Similarity searching

## Abstract

**Background:**

Ligand-based *in silico* target fishing can be used to identify the potential interacting target of bioactive ligands, which is useful for understanding the polypharmacology and safety profile of existing drugs. The underlying principle of the approach is that known bioactive ligands can be used as reference to predict the targets for a new compound.

**Results:**

We tested a pipeline enabling large-scale target fishing and drug repositioning, based on simple fingerprint similarity rankings with data fusion. A large library containing 533 drug relevant targets with 179,807 active ligands was compiled, where each target was defined by its ligand set. For a given query molecule, its target profile is generated by similarity searching against the ligand sets assigned to each target, for which individual searches utilizing multiple reference structures are then fused into a single ranking list representing the potential target interaction profile of the query compound. The proposed approach was validated by 10-fold cross validation and two external tests using data from DrugBank and Therapeutic Target Database (TTD). The use of the approach was further demonstrated with some examples concerning the drug repositioning and drug side-effects prediction. The promising results suggest that the proposed method is useful for not only finding promiscuous drugs for their new usages, but also predicting some important toxic liabilities.

**Conclusions:**

With the rapid increasing volume and diversity of data concerning drug related targets and their ligands, the simple ligand-based target fishing approach would play an important role in assisting future drug design and discovery.

## Background

For many decades, the drug discovery and development have been directed by the idea of ‘one drug–one target–one disease’. The paradigm is shifting since many drugs elicit their therapeutic activities by modulating multiple targets, as indicated by the polypharmacology [1-3]. However, multi-target interactions are either unknown or insufficiently understood in most cases, which inspired many efforts to predict and characterize drug–target associations.

Use of *in silico* tools to predict targets of small molecules has drawn more and more attentions in recent years. These predicted drug targets can be divided into two types: I) unexploited novel drug targets that can be used alone or with other drugs in combination chemotherapy treatment [3]; II) existing drug targets that provide new uses and indications for existing drugs [4]. One of the most prominent examples for drug repositioning is Sildenafil, which was initially developed for use for hypertension and angina, and then repositioned for the treatment of male erectile dysfunction [5]. Other notable drug repositioning examples include Memantine [6], Buprenorphine [7], Requip [8,9], Colesevelam [10], and so on. Numerous computational strategies for target fishing have been published. These studies enable researchers to deepen the understanding of the bioactive space of new chemical entities, which provide an efficient way in designing ligands with favorable pharmacological and safety profile. Generally, available target fishing approaches fall into the following two major categories:

### 1. Target-based Methods

Target-based methods use the information of target proteins, which includes molecular docking, similarity comparison of protein sequence or binding pocket, and so on. For example, INVDOCK [11] and TarFisDock [12] screen a query small molecule against a panel of predefined target protein structures whereby putative targets are sorted by docking score [13]. This approach has been demonstrated to be useful in target identification, and some of the predicted results have been verified by bioassay and crystallographic studies [14,15]. Although significant improvements have been made in this area, there are still practical limitations for target structure-based approaches, such as unavailable crystal structures (especially for most trans-membrane proteins), high false positive rate, the choice of an appropriate scoring function and high requirement of computational resources [16]. To circumvent these issues, several target-based methods relying on the analysis of existing drug-target interaction data have been developed. For instance, Luo *et al.* developed a web server DRAR-CPI to identify drug repositioning and adverse drug reactions by mining chemical–protein interactome [17]. Milletti *et al.*[18] and Wang *et al.*[19] predicted polypharmacology by comparing the structural similarity of binding sites. Recently, Jacob *et al.*[20] and Wang *et al.*[21] constructed chemogenomics approaches for qualitatively predicting ligand-protein interaction that only require the primary sequence of proteins and the structural features of small molecules. These approaches transform the target fishing problem to a machine learning problem in the ligand–target space. Though potentially useful, they are sensitive to how a given target protein or ligand-protein pair is represented by descriptor vectors, and have a limited application domain defined by their training set range.

### 2. Ligand-based Methods

Ligand-based methods simplify the problem to a similarity searching problem, and only use ligand information to predict target. Compared with the structure-based approaches, ligand-based approaches do not rely on the complete knowledge of ligand-target interaction mechanisms and requires relatively low computational cost. Based on how a given ligand is represented, these methods can be divided as 2D fingerprint, molecular shape, pharmacophore, and bioactivity spectrum-based, *etc*.

Chemically similar drugs often bind biologically relevant protein targets. To uncover the pharmacological relationships among proteins, Keiser *et al.* developed a statistics-based chemoinformatics approach called similarity ensemble approach (SEA) [22], in which each target was represented solely by the structures of its set of known ligands. SEA has been applied to quantitatively identify pharmacological links between targets by the similarity of the ligands bind to them, expressed as expectation values (E-value). It was further successfully applied to large-scale test for drug repurposing [23]. Furthermore, three dimensional (3D) molecular shape descriptors have turned out to be especially successful in describing and comparing molecular profiles. Abdul Hameed *et al.* developed a novel approach by comparing shape similarity using program ROCS [24]. In their approach, target profiles were generated for a given query molecule by computing the maximal 3D-shape and chemistry-based similarity to the collection of drugs assigned to each protein target [25]. Pharmacophore, like molecular docking, can also be reversely used for *in silico* drug target identification. Recently, Liu *et al.* reported a free web interface PharmMapper that uses pharmacophore to predict protein targets for small molecules [26]. This approach automatically performs reverse mapping against the deposited pharmacophore models and outputs the top ranked hits. With the rapid growth in bioactivity data of small molecules and their targets, it is possible to employ the information to infer targets for drugs or bioactive compounds. Cheng *et al.* developed an approach named bioactivity profile similarity search (BASS), for associating targets to small molecules by comparing the bioactivity profiles that are derived from the NCI-60 cell lines [27].

A notable strategy for similarity searching is data fusion (DF) that utilizes multiple reference structures to search against a database. A DF process is to combine the information provided by multiple independent sensors in order to make judgments on an event, which was firstly proposed by Peter Willett and his coworkers [28]. Afterwards, Whittle *et al*. [29] and Hert *et al.*[30] used 2D fingerprint similarity ranking with DF for virtual screening, and demonstrated its effectiveness over conventional similarity searching in scaffold-hopping searches for structurally diverse sets of active molecules [31]. Due to its high searching quality and low computational cost, this approach is especially fit for the exponential growth in biological data.

Although many advances have been made over the last decades, drug target prediction is still a very challenging task as reflected by the low clinical target validation success rate. The reasons are manifold, yet what poses the greatest difficulty might be the amount of protein targets and known active small molecules. For example, the current version ChEMBL database (version 17) [32] contains 12,077,491 bioassay data for 9,356 targets and 1,324,941 compounds. Such data collection is so large and complex that it becomes difficult to process using traditional molecular modeling process and target-ligand interaction applications. In this regard, we may consider the target fishing as a ‘big data’ problem. As defined by Donglas Laney [33], big data problems mainly have three aspects of features of data growth, *i.e.* having increasing volume (amount of data), velocity (speed of data in and out), and variety (range of data types and sources). For target fishing, vast amount of data in various measurements (K_i_, K_d_, IC_50_, inhibition rates and so on) are being generated daily from different sources, with the fast development of many high-throughput bioassay systems. Given the data of these features, we need firstly make a practical trade-off between the amount of employed data and the complexity of models. Though under debate, it has been widely realized that using more data is more beneficial, because it provides the contextual richness in data and does not rely on unproven assumptions and weak correlations. From this aspect, we may argue that more emphasis should be placed on the data set used for target fishing, instead of developing algorithms that are more sophisticated. In this study, we try to address the target fishing problem from the ‘big data’ perspective. A large reference ligand library is first established, with each ligand set to represent a single target. Here, the DF strategy is adopted to calculate the highest K similarity scores (or their average value) between the query and the ligands sets in reference library, using Tanimoto coefficients (Tc) of ECFP4 fingerprints. The value of K can be 1, 3, and 5 (denoted as Max, 3NN, and 5NN, respectively), and the average fusion similarity is a centroid score, which is described in Methods section. The target profile of a query chemical is then provided according to the ranked fusion scores. The performance of this scheme is tested on two test sets, and a further validation is made to identify new (off-) targets and hERG related toxicity. The aim of the study is to benchmark the target fishing capability by using a simple ligand-based similarity searching approach, in the meantime, by employing the available data as much as possible.

The SEA approach represents a notable recent advance in identifying protein targets. Here, a locally implemented SEA approach was run in parallel with our approach for accuracy assessment, of which the E-value was used to rank potential targets [22]. We perform this comparison because both SEA and our approach use active ligand set to represent target, and use 2D fingerprint based similarity to obtain the score of a target (The SEA approach can be considered as a data fusion scheme, where the score of a target is normalized by the size of its ligand set). It should be pointed out that SEA requires that the product of the ligand set sizes is not less than 100 to guarantee statistically reliable result [34]. It means that the current SEA is not appropriate for fishing targets without sufficient reference ligands. Nevertheless, its result can serve as a control to see how existing approaches perform on the current data set.

## Results and discussion

**Figure 1:**
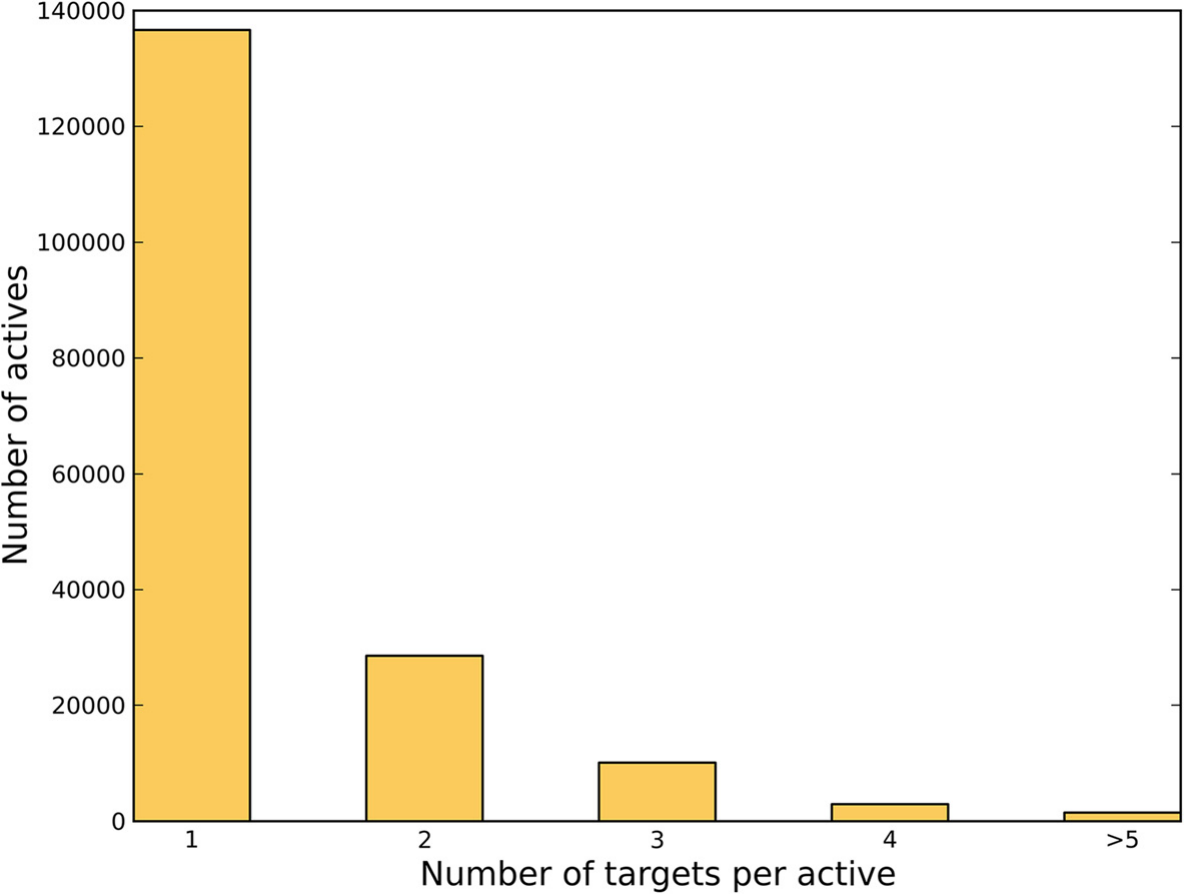
Plot to show the distribution of the number of active ligands against the number of targets per ligand.

Drug related targets (DRTs) and their ligands are favorable sources for analyzing target-ligand interaction and understanding polypharmacological effects of drugs. As described in Methods section, one reference library containing DRTs with active ligand set and two validation sets were compiled for this study. Table 1 summarizes the number of compounds and targets of each set. We further analyzed the polypharmacological profile of the ligands in the reference library. As shown in Figure 1, most active ligands have only one single target, while there are also significant amount of ligands having two or three targets. The number of ligands having many targets is small, and 1,512 ligands have the number of targets greater than five.

**Table 1:** Statistics of the data sets used as reference ligand library and for external validation sets

	**Data Sources**	**Target**	**Actives (or drugs)**	**Pairs**	**Ref.**
**Reference library**	BindingDB	533	179807	246053	[40]
**External validation sets**	DrugBank	455	711	7917	[41]
	TTD	255	476	1084	[43]

Given a ligand that has *m* experimentally verified targets, a target fishing scheme yields *n* predicted targets for the ligand (*i.e.*, the top ranked *n* targets), we used the following evaluation metrics to measure the performance of the scheme: Precision (*PR*_*n*_), Recall (*RE*_*n*_), F-measure (*F*_*n*_) [35], and the uninterpolated precision (*PR’*) [36]. *PR’* is given by the averaged precision values *PR*_*i*_ from the ranking places 1 to *m*. Here, *m* for a query ligand is the number of its interacting targets. The detailed definitions of these terms are provided in the Methods section.

### 1. Ten-fold cross-validation

The 10-fold cross validation was performed to determine the parameter K for the nearest neighbor to fuse and to evaluate the effectiveness of the fusion strategy when only a small part of the set was used as reference. In the validation, the overall reference set was randomly split into ten parts. For the ligands of each part, their targets were predicted using the ligands and the targets information of the rest 9 parts. The performance achieved for each part was recorded, and the average *PR’* value was used to evaluate the four fusion schemes as well as SEA for comparison. Since SEA calculates the set-wise similarity among ligand sets, it would not be statistically reliable if the ligand sets comprising fewer than ten ligands. In practice, it is suggested that the product of the set sizes should be higher than 100 [22,34]. In the case of target fishing, the set-wise similarity is typically calculated between a single query ligand and a reference ligand set. So in order to perform a comparison, we did another test for SEA only using the reference targets whose ligands are equal to or more than 100. Altogether, 292 targets with 173,862 ligands (23,6986 pairs) were retained as another unique reference set to test SEA performance. As outlined in Table 2, Max scheme performs a little worse than 3NN and 5NN schemes. Both 3NN and 5NN show very close results and 3NN slightly outperform the others. A possible explanation is that 5NN includes some ligands that are not very similar to the query. Instead, the first three neighbors of a query in reference ligand set may better represent the corresponding target, and discriminate among other optional targets. Moreover, it is clear that Centroid score is less effective than other KNN schemes. In our experiment, SEA performs a bit worse than KNN fusion. As expected, KNN strategy is able to determine the target of small molecules with significant accuracy and robustness in internal cross validation.

**Table 2:** **The result (*PR’*) of 10-folds cross-validation on the reference set**

	**Max**	**3NN**	**5NN**	**Centroid**	**SEA**	SEA*
**Mean**	0.927	0.950	0.947	0.228	0.628	0.717
**S.E.**	0.002	0.002	0.002	0.002	0.001	0.001

*Only the targets containing more than 100 reference ligands were considered in the validation.

*PR’* is given by the averaged precision values *PR*_*i*_ from the ranking places 1 to *m*. Here, *m* for a query ligand is the number of its interacting targets.

**Figure 2:**
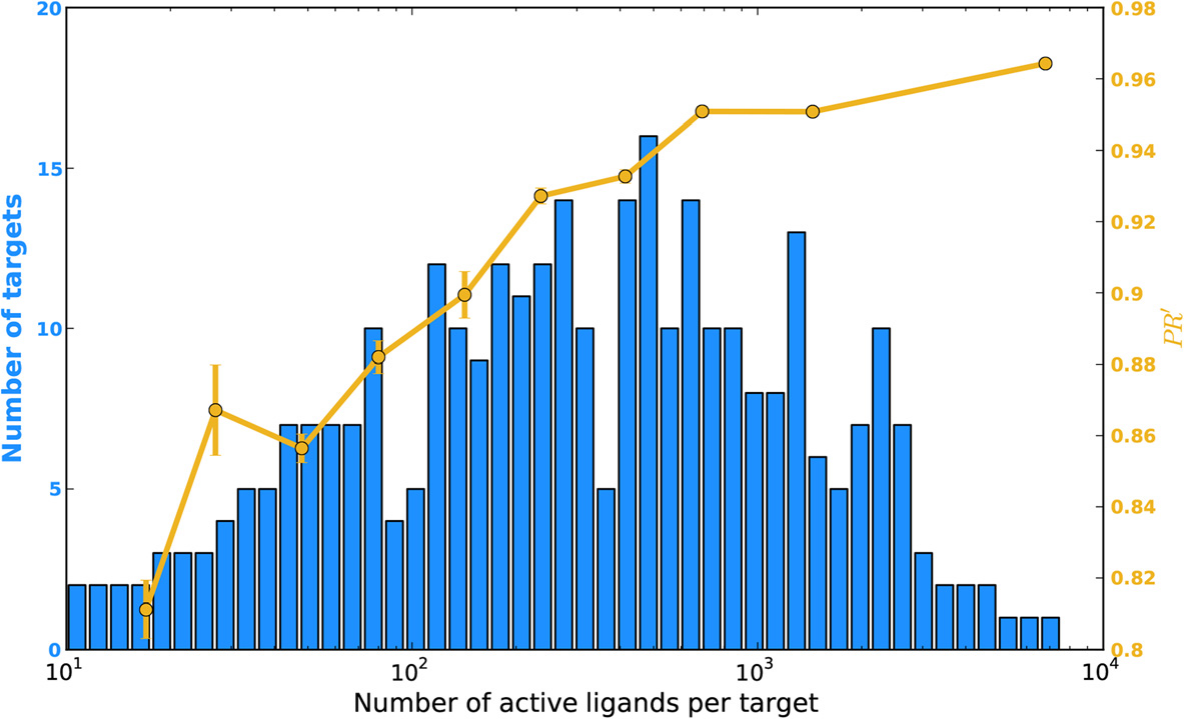
**Plot to show the distribution of average *PR’ *against the targets with increasing number of reference ligands.***PR’* is given by the averaged precision values *PR*_*i*_ from the ranking places 1 to *m*. Here, *m* for a query ligand is the number of its interacting targets.

Since the targets here are represented by their reference ligands, the predictive ability relies on the representativeness and diversity of reference library. Figure 2 displays a bar plot of the number of active ligands for all the targets in the reference set. Among all the 533 targets having more than 10 active ligands, 292 of them have more than 100 active ligands and 72 of them have more than 1000 active ligands. These 533 approved drug targets cover the major members of clinical therapeutic protein receptors, enzymes and disease related targets. With the amount of bioassay data growing, our reference library can be easily extended to incorporate more ligand-target interaction data. Then, we would like to know how 3NN behaves on targets with different numbers of reference ligands. We studied the *PR’* in 10-CV by grouping the reference targets evenly into 10 bins according to the amount of its ligands. The *PR’* of a bin is defined as the average *PR’* score for all the targets in that bin. The yellow line shown in Figure 2 depicts how the *PR’* varies across targets with ascending number of reference ligands. As the number of reference ligands increases, *PR’* increases and the error bar decreases, suggesting 3NN tends to perform better for the targets with a large number of reference ligands. Overall, the *PR’* scores range from 0.8 to 0.96, demonstrating that 3NN has excellent accuracy for fishing targets with adequate reference ligands. At the same time, we may also find that the approach showed a reasonable performance on targets with a small number of reference ligands. As shown in Figure 2, the majority of targets collected in our reference set have adequate reference ligands ranging from 10^2^ to 10^3^, which ensured high predictive ability of the approach.

### 2. The performance of 3NN with increasing size of reference Set

**Figure 3:**
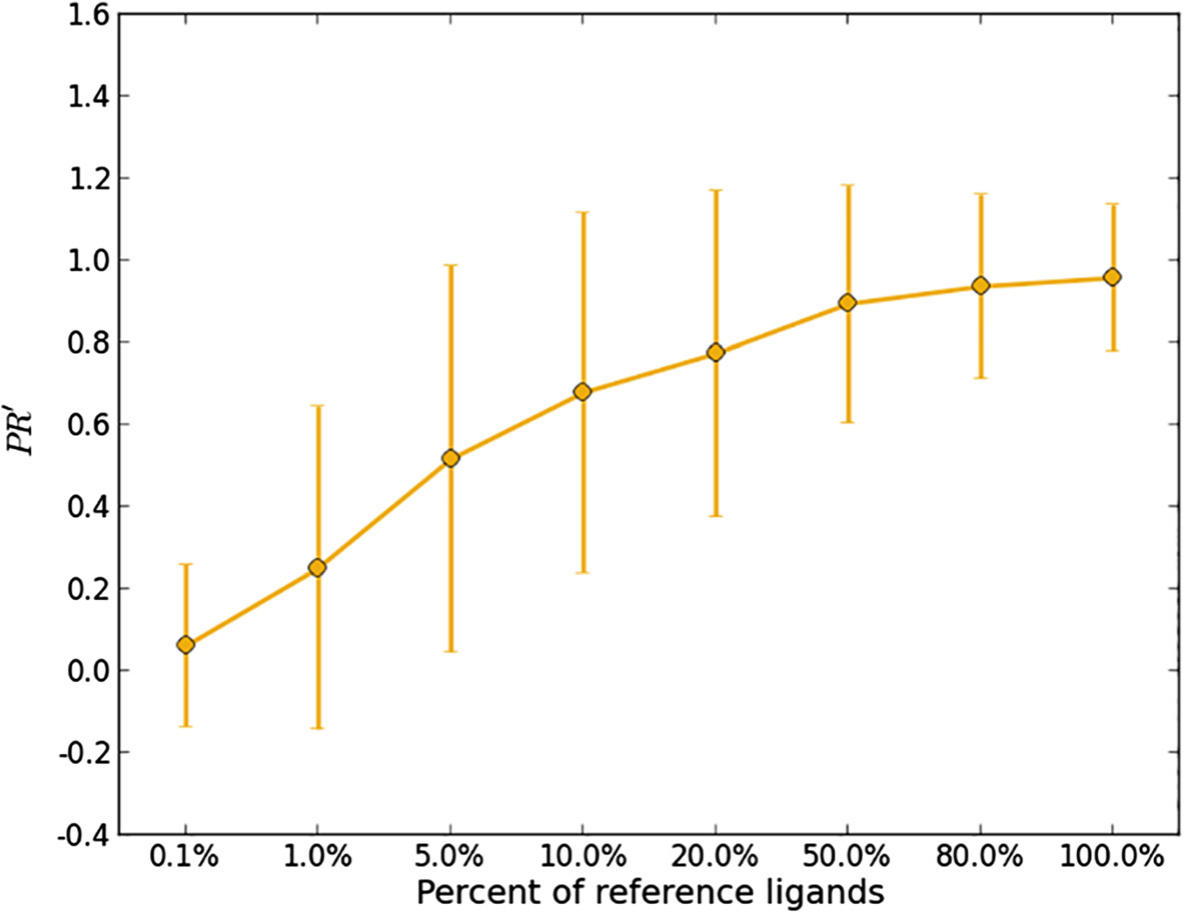
**Plot to show the change of *PR’ *** **with increasing percent of the reference ligands in use.**

With increasing number of active ligands available in various databases, we want to revisit target prediction in the context of a ‘big data’. The reference set we collected contains more than a hundred thousand active ligands and it will definitely grow in the future. Therefore, we would like to investigate the performance change of 3NN as more reference compounds become available. As every target is represented by a set of active ligands, we created a test set of 2665 ligands by randomly picking five ligands from each of the 533 targets. The remaining reference ligands were used as the reference set. In order to study 3NN on different sizes of reference sets, a total number of eight reference sets were made by randomly sampling 0.1%, 1%, 5%, 10%, 20%, 50%, 80% and 100% from the remaining reference ligands. Then, we ran 3NN utilizing each of reference sets and record their *PR’* values. The experiment was repeated five times and the overall result was depicted in Figure 3. When only 0.1% information is used, the average *PR’* is close to 0 with a small SD of 0.2, suggesting that most of the targets cannot be identified by the small reference set. The *PR’* is gradually increasing as more reference information involved. When 10% references were used, the average *PR’* is more than 0.6 but the error bar is relatively larger (SD = 0.44). It suggests that the prediction accuracy of 3NN may reach a high level if the related reference set is of considerable volume, but the variance of prediction is also large, which is also the result that would occur in most cases for similarity-based approaches. When 50% reference ligands were used, we may find that the *PR’* had a notable increase to 0.89 and SD reduced to 0.29, which indicates that more targets could be identified for test molecules. It is also worth noticing that the line flattens and the error bar decreases when more than half of the references were used, which shows that there is only marginal gain in prediction performance (average *PR’*) if the related reference set is of a sufficient large volume. Finally, by using the total reference set, the average *PR’* and SD are 0.96 and 0.18, respectively. The test demonstrates that the more prior knowledge may not only improve the prediction accuracy of 3NN fusion strategy in target predicting, but also reduce its prediction variance.

**Figure 4:**
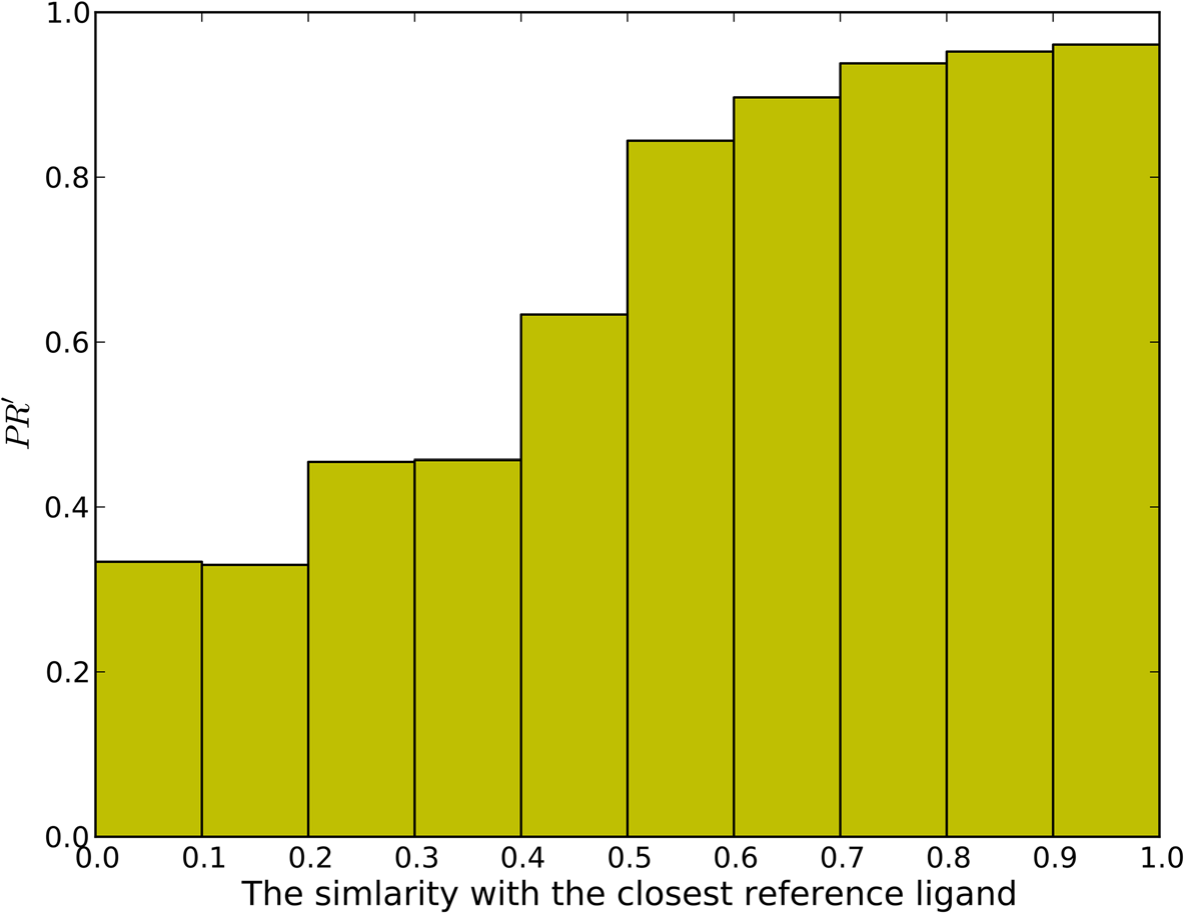
**The bar plot showing the variation of *PR’ *versus the similarity of a query to its closest neighbor in its corresponding reference ligand set.** This analysis is based on the test set containing 2665 query ligands.

Given a query compound, it is interesting to investigate how similar ligands the reference set contains and whether the similarity will affect target predicting. For the test set containing 2665 compounds, we checked the variation of *PR’* versus the similarity of a query to its closest neighbor in its corresponding reference ligand set, as shown in Figure 4. On the one side, we may find the 3NN model always gave a high *PR’* value if the query can find close neighbors. This observation suggests that a sufficiently large and diverse reference library is important for the predicting accuracy, which explains why the target fishing problem should be addressed from a “big data” perspective. On the other side, we may also notice that the 3NN model is robust, as the *PR’* reaches 0.65 for those have 0.4 ~ 0.5 similarity scores. It means that the model is still useful when the query can only retrieve some moderately similar ligands.

In general, 3NN achieves high *PR’* in the internal validation, which is partially attributed to the close analogues that exists in the both test and reference sets. To assess the performance of the approach further on practical cases, two external tests are performed and analyzed in the following section.

### 3. Predicting targets for approved drugs from drug bank and TTD

Many drugs from a wide range of therapeutic areas have more than one interacting targets, and the multiple on-target and off-target bindings are essential for their efficacy and side effects. For example, the number of reported interacting targets for the drugs treating central nervous system disorders is even up to 64 in our validation set. We compared 3NN and SEA for target identification for these multi-target drugs. For each test compound, we considered the top 20 predicted targets, in terms of the metrics including *PR*_*n*_, *RE*_*n*_, *F*_*n*_ and *PR’*. The averaged results of the 711 drugs presented in the DrugBank and 476 drugs in TTD are reported.

**Figure 5:**
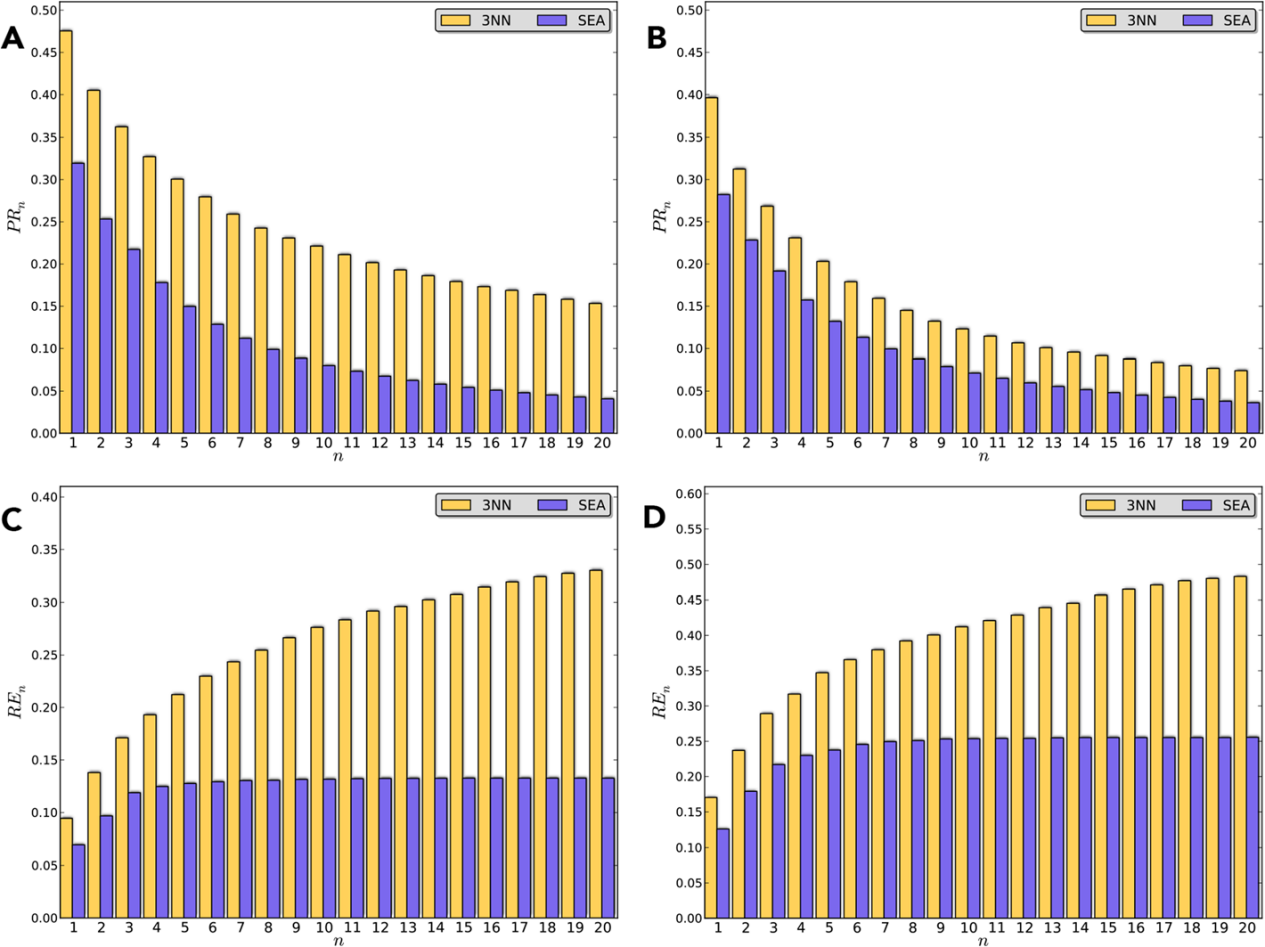
**Comparison of *PR***_***n ***_**by 3NN and SEA for the drugs from: (A) DrugBank set and (B) TTD set; Comparison of *RE***_***n ***_**by 3NN and SEA for the drugs from: (C) DrugBank set and (D) TTD set.**

Figure 5(A-D) show the performance changes of the top 20 predictions for the DrugBank and TTD ligands. Clearly, the 3NN scheme performs better in the validation, of which all the metrics are consistently higher than those of SEA. As depicted, the results of the two approaches show a similar trend except for the following minor differences: For the 3NN scheme, gradual decreasing *PR*_*n*_ and increasing *RE*_*n*_ are observed, and the changes are more significant when *n* is small. For SEA, *PR*_*n*_ decreases more quickly, and its value is close to zero when *n* is larger than 10. *RE*_*n*_ of SEA shows a different appearance, where it only exhibits increase when *n* is less than 5, and then leveled off. These results mean that SEA can only identify the “true targets” in a few top ranked predictions, and further increasing the number of predictions will not yield more “true targets”. In contrast, 3NN is able to retrieve more experimentally observed targets as it allows more predictions, demonstrating its advantage in addressing the polypharmacology of drugs.

**Figure 6:**
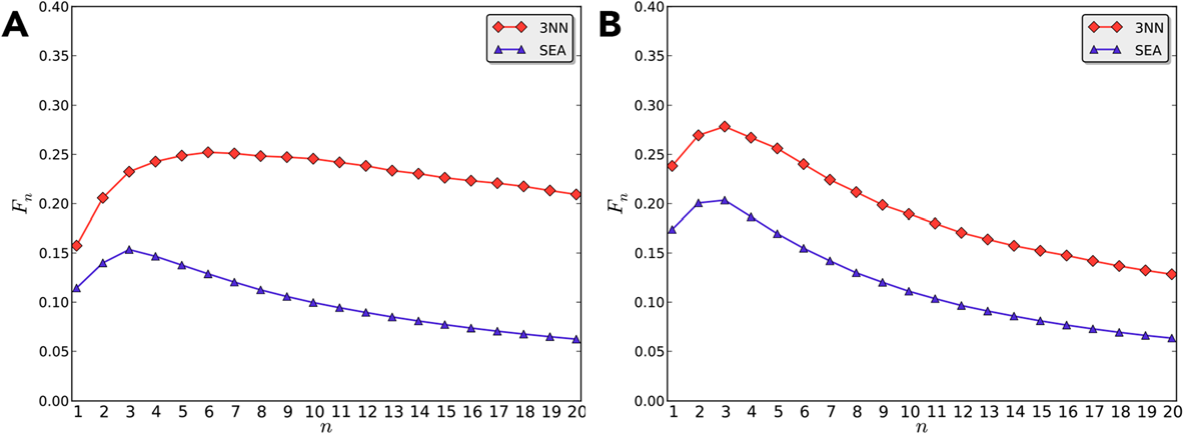
**Comparison of *F***_***n ***_**by 3NN and SEA for the drugs from: (A) DrugBank set and (B) TTD set.**

Figure 6(A) and 6(B) show the *F*_*n*_ curves of 3NN and SEA for drugs from DrugBank and TTD, respectively. There are a few differences between these two validation sets DrugBank and TTD. TTD mainly contains the primary targets directly related to the therapeutic actions of approved drugs, while DrugBank collects more comprehensive potentials targets. In addition to the targets that confer the desired pharmacological effect, DrugBank also contains other targets including metabolism enzymes, carrier and transporters. These targets usually account for the side effects or drug-drug interactions. A comparison of these two sets shows that 64% drugs in TTD also present in Drugbank. Therefore, we may consider TTD as a subset of DrugBank, in which TTD only includes pharmacological targets, and DrugBank includes more comprehensive interaction targets. Further inspection of Figure 6 reveals that the 3NN for DrugBank displayed a different pattern on these two sets. As shown in Figure 6(A), the *F*_*n*_ of 3NN achieves its maximum value for DrugBank when *n* equals to 6 (the vertex of the curve). Clearly, we may find that the *F*_*n*_ curves of 3NN are consistently higher than SEA, suggesting its higher performance on predicting drug targets.

From Figure 6(B), we may also notice that 3NN and SEA show a similar tendency on TTD, of which the *F*_*n*_ curves rapidly decline when *n* > 3. It suggests that the therapeutic targets can be well identified in the top three predictions, and considering more targets ranked outside the top three would result in a significant number of false predictions. However, if one aims to predict non-therapeutic targets as well, the prediction rank list should be extended. As shown in Figure 6(A), the decline of 3NN is still slow when *n* > 6. Another point of notice is that for both 3NN and SEA the maximum *F*_*n*_ value obtained on TTD is higher that on DrugBank. This observation suggests that therapeutic targets could be more reliably predicted. One of the possible reasons is that the therapeutic targets usually form specific interaction with their corresponding drugs with high affinities. However, the non-therapeutic targets, *e.g.* CYP450s, may exhibit enormous promiscuity, and they interact with a huge range of structurally unrelated ligands. The weak and non-specific interactions may lead to inferior performance on predicting drugs interacting with these targets. More details about this test are provided in Additional file 1: Table S1 and Table S2.

Alternative target fishing methods include 3D similarity searching methods as well as those based on machine learning. The 3D similarity searching methods rely on the generation of active conformations for both references and queries, which are difficult to obtain for some flexible compounds and involve high computational cost. For the machine learning methods, both known active and inactive molecules should be present to form a training set. However, the true inactive data are hardly available in most public databases, thus significantly restricted their usages in target fishing. In comparison, the 2D similarity searching methods only require the positive data and the chemical fingerprints fast to compute, making it an efficient method for large-scale target fishing.

### 4. Identification of New (Off-) target-drug interactions

From the previous analysis, we may notice that 3NN DF scheme based on a large reference set is suitable for the ligands with multiple targets. It is therefore interesting to investigate its performance on identifying the new and off-targets from the experimentally verified drug-target associations. To this end, we tested 3NN using Keiser’s data that were previously used to verify the prediction of SEA [23]. The first test set includes within-boundary predictions for 10 GPCR drugs and cross-boundary predictions for 4 non-GPCR drugs, and the second set includes 32 drugs with 39 off-targets associations.

Table 3 shows the 3NN rankings of the new targets for the drugs. We noticed that 62 out of 65 new drug-target associations can be “hit” at the top 20 predictions, and most of the targets are predicted in top 1 ~ 6. This result is consistent with the vertex shown in Figure 6(A) that 3NN could achieve a good predictive ability in top 1 ~ 6. It also means that new (off-) targets could be successfully identified in nearly top 1% of the full set of 533 targets. Only a few experimentally verified target-ligand interactions were ranked outside of top 20. For example, the interaction of β_1_ adrenergic receptor with Prozac and Paxil was ranked at the 39th and 75th places by 3NN, respectively. These two drugs were predicted to interact with β adrenergic receptor by SEA, and later experiments revealed that they are medium-potency blockers of β_1_ subtype (*i.e*., K_i_ = 4.4 μM for Prozac and 10 μM for Paxil). Since our DRT reference set mainly focuses on strong binders to a specific protein, the low rankings of the target for these two drugs may be partially attributed to their low binding affinities to the target, which are close to the threshold for selecting ligands in our reference set. Another important feature of our DRT reference set is it includes more target members that were categorized according to their sequence similarities. Compared with the reference set of SEA, our DRT reference set specifies the three subtypes of β adrenergic receptor, hence not requiring a separate assay for each one. Actually, for Prozac and Paxil, their interactions with β_1_ were ranked highest among the three subtypes, which are consistent with Keiser’s assay results.

**Table 3:** **Target ranking results of the 3NN scheme on the novel drug-target association set of Keiser** ***et al*.**[23]

**Drug**		**Pharmacological Action**	**New (off-) targets**	**3NN Ranking**
**New aminergic GPCR targets**	Sedalande	Neuroleptic	αe adrenergic blocker	3
			5-HT1D antagonist	1
	Dimetholizine	Antihistamine; antihypertensive	αn adrenergic blocker	5
			5-HT1A antagonist	1
			D2 antagonist	2
	Kalgut	Cardiotonic	βa adrenergic agonist	1
	Fabahistin	Antihistamine	5-HT5A antagonist	3
	Prantal	Anticholinergic; antispasmodic	δntichol agonist	1
	N,N-dimethyltryptamine	Serotonergic hallucinogen	5-HT1B agonist	1
			5-HT2A agonist	2
			5-HT5A antagonist	13
			5-HT7 modulator	11
	Doralese	Adrenergical blocker; antihypertensive; antimigraine	D_4_ antagonist	6
	Prozac	5-HT reuptake inhibitor; antidepressant	β adrenergic blocker	39
	Motilium	Antiemetic; peristaltic stimulant	αe adrenergic blocker	6
	Paxil	5-HT reuptake inhibitor; antidepressant	β adrenergic blocker	75
**New cross-class targets**	Xenazine	αe (transporter)	αe adrenergic receptor (GPCR)	6
	Rescriptor	HIV-1reverse transcriptase (enzyme)	H4 receptor (GPCR)	4
	Vadilex	NMDAR (ion channel)	μMDAR (i receptor (GPCR)	16
				4
			5-HTT	14
			SERT(transporter)	
	RO-25-6981	NMDAR (ion channel)	5-HTT	5
			SERT (transporter)	13
			D4 receptor (GPCR)	6
			noradrenaline transporter(transporter)	13
			κoradren receptor (GPCR)	20
**Other off-targets**	Amisulpride	Antipsychotic	D2 Antagonist	1
	Aripiprazole	5-HT1A Agonist	D3 Antagonist	1
		5-HT2A Antagonist	D2 Antagonist	1
		Alcohol Deterrent Antiamyloidogenic Agent Antipsychotic Treatment of Cocaine Dependency		
	Benperidol	Antipsychotic	5-HT2A Antagonist	2
			D4 Antagonist	10
	Benzoclidine	Antihypertensive Anxiolytic	M3 Antagonist	2
	Bromperidol	Antipsychotic	5-HT2A Antagonist	6
	Cabergoline	Prolactin secretion inhibitor	Dopamine Agonist	2
			Adrenoceptor (renoceptornist	2
			5-HT1D Agonist	1
	Captopril	ACE Inhibitor Antihypertensive Cardiotonic	Leukotriene A4 Hydrolase Inhibitor	6
	Carbacyclin	Antithrombotic	Prostaglandin	2
	Carvedilol	Antianginal Antihypertensive	Adrenergic (β) Blocker	1
	Enrofloxacin	Antibacterial Quinolone	DNA gyrase	1
	Fluanisone	Neuroleptic	5-HT2A Antagonist	8
	Hexoprenaline	Bronchodilator	Adrenergic (β) Agonist	1
	Linezolid	Antibacterial Oxazolidinone	MAO A Inhibitor	2
	Loratadine	Antihistaminic Inflammatory Bowel Disease	Farnesyl Protein Transferase Inhibitor	382
		Rhinitis		
	Melperone	Neuroleptic	5-HT2A Antagonist	1
	Metergoline	Antimigraine Vasodilator	Adrenoceptor (renoceptor Vas	2
	Naftopidil	Prostate Disorders	5-HT1A Antagonist	7
			α- adrenergic Blocker	2
	Naringenin	Antiulcerative Enzyme inhibitor Enzyme inhibitor (Histidine decarboxylase)	Xanthine Oxidase Inhibitor	16
	Nuvenzepine	Antiulcerative	M2 Antagonist	1
	Pimozide	Antipsychotic	Anticholinergic, Ophthalmic	3
			5-HT2A Antagonist	16
	Rabeprazole	Antisecretory (gastric acid) Antiulcerative	H^+^/K^+^-ATPase Inhibitor	1
	Rispenzepine	Antispasmodic	M2 Antagonist	1
	Tetrabenazine	Anxiolytic	D1 Antagonist	3
	Tetraminol	Antihypertensive Vasodilator	Adrenoceptor (renoceptorsive	2
	Urapidil	αr adrenergic Blocker Antihypertensive	5-HT1A Antagonist	2
	Cinitapride hygrogen tartrate	Antiulcerative Stimulant, Peristaltic	5-HT4 Agonist	1
	Lisuride maleate	Antiparkinsonian Dopamine Autoreceptor Agonist Prolactin Secretion Inhibitor	Adrenoceptor (renoceptorlact	1
	Methylphenidate	Adrenergic Agents Adrenergic Uptake Inhibitors Central Nervous System Stimulants Dopamine Agents Dopamine Uptake Inhibitors Sympathomimetics	M3 Antagonist	3
	Pergolide mesylate	Antiparkinsonian, Dopamine Agonist	5-HT1D Agonist	2
			Adrenoceptor (renoceptorstni	1
	Propafenone hydrochloride	Antiarrhythmic	βntiarrhythm blocker	3
	Terbinafine hydrochlorid	Antifungal	Squalene Epoxidase Inhibitor	1
	Urapidil	αr adrenergic Blocker Antihypertensive	D2 Antagonist	3

*Abbreviations*: *5-HT* Serotonin, *D* Dopamine receptor, *HIV* Human Immunodeficiency Virus reverse transcriptase, *H* Histamine, *NMDAR* N-methyl-D-aspartate receptor (glutamate receptor), *SERT* serotonin transporter, *M* Muscarinic acetylcholine receptor.

### 5. hERG toxicity prediction

Off-target interactions are typically related to adverse drug effects, among which a prominent example is the interaction of numerous compounds with hERG, a potassium ion channel expressed in the heart and in nervous tissue. In the past decade, a frequent cause of the withdrawal of the marketed drugs has been the potentially fatal arrhythmia that is induced by a blockage of hERG channels [37,38]. In this study, we further investigated the hERG-related off-target prediction using the 3NN target fishing scheme. Table 4 list nine approved drugs withdrawn from the market due to hERG toxicity [37]. For seven of these drugs, their interactions with hERG were predicted in the top 20 list. For three drugs, terfenadine, Sparfloxacin, Droperidol, their interactions with hERG were even ranked at the first place. For comparison, the rankings of therapeutic targets of these drugs were also listed. We notice that all the on-target interactions of 9 drugs fall in the top 20 list. These results highlight the usefulness of the 3NN scheme on identifying both on and off-targets. Particularly, the high ranking of hERG as a potential interacting target of a query compound may serve as a hERG toxicity alert for further safety investigation.

**Table 4:** Target ranking results of the 3NN scheme on nine drugs with hERG toxicity

**Drug**	**Therapeutic target**	**3NN ranking (TT)**	**3NN ranking (hERG)**
**Astemizole**	H1 receptor	3	2
**Cisapride**	5-hydroxytryptamine 2A/3/4 receptor	5	6
**Sertindole**	D2 receptor	1	2
	5-hydroxytryptamine 2A/2C/6 receptor	4	
	α-hydroxytr adrenergic receptor	5-7	
**terfenadine**	Histamine H1 receptor	2	1
	Potassium voltage-gated channel subfamily H member 2	1	
	M3	5	
**Sparfloxacin**	DNA topoisomerase 4 subunit A	2	1
	DNA gyrase subunit A	3	
	DNA topoisomerase 2α	6	
**Droperidol**	D2 dopamine receptor	3	1
	α2 adrenergic receptor	5	
**levomethadyl Acetate**	μAceta opioid receptor	1	27
	Neuronal acetylcholine receptor	3	
**Lidoflazine**	Calcium channel	1	21
**Terodiline**	Calcium channel	47	4

*Abbreviations*: *TT* Therapeutic target, *H* Histamine, *D* Dopamine receptor, *M* Muscarinic acetylcholine receptor.

## Experimental

### 1. Reference set preparation

While there are many public databases (ChEMBL [39], BindingDB [40], PubChem, *etc.*) storing bioactive small molecules and target information, there are no special collections for ligands of DRTs. Here, we build a collection of the active ligands from BindingDB for FDA-approved drug targets from DrugBank [41]. The detailed procedures for the data set preparation are as follows:

(I) DrugBank provides a list of FDA-approved drug targets, among which all protein sequences of drug targets of small molecules were downloaded. Sequences of protein targets deposited in BindingDB were used to create a local BLAST database with NCBI blast [42].

(II) The downloaded sequences from DrugBank were used to perform similarity search against the local BLAST database, to find drug-target related targets in BindingDB and to retrieve their interacting ligands. Using an E-value threshold (1E-50), we obtained target mapping between BindingDB sequences and drug target sequences from DrugBank. A protein target of BindingDB exhibiting high homology with any of the drug targets was considered as a potential drug target.

(III) The ligands were further filtered to eliminate those with weak binding affinity to a specific protein. The threshold for “active” ligand was set as IC_50_, K_i_, K_d_ or EC_50_ < 10 μM, or ΔG <28.53 kJ/mol.

(IV) The above retrieved protein targets were redundant (*i.e.* there are identical proteins with different names), and some of them are highly homologous to each other (*e.g.* mutants or from different source organisms). To address the issue, we combined the proteins showing high sequence similarities by another round of BLAST searches, with a more stringent E-value threshold of 1E-120. All the active ligands of a “combined” protein target were pooled together. The resulting database contained 725 targets in all.

(V) To ensure every target has a certain amount of ligand representatives, we filtered those targets whose active ligands were less than or equal to 10. At last, our curated database covers 533 targets with 179,807 active ligands in total. Approved drugs are used as an independent test set for additional validation.

This established chemical reference library is organized according to DRTs, and each of them is represented by a set of corresponding active ligands. In our reference library, the ligand set contains unique ligands for each target. All the data preparation procedures are performed with in-house Python scripts. The reference library is designed to enable further updating by adding new target-ligand interaction data.

### 2. Validation sets preparation

The following two datasets were used to test the target predicting performance of different approaches, including approved drugs from Therapeutic Target Database (TTD) [43], and approved drugs from DrugBank 3.0 [41]. These datasets contains drug or drug-like compounds and their protein target sequences. For each set, the small molecules existing in the reference library were firstly removed, and the sequences were mapped onto DRTs by similarity searching against the local BLAST database mentioned above.

## Conclusions

With the rapid advancement of high-throughput screening technology, the shear amount of bioassay data is so huge and increasing so fast that many traditional frameworks encounter difficulties on launching a large campaign of target fishing. The exploration of more efficient approach in the context of ‘big data’ is needed for the challenging task. In this study, we exploited a simple scheme using 2D fingerprint similarity ranking with a DF strategy to predict drug-relevant targets based on a reference library containing 533 targets with 179,807 active ligands. This scheme exhibits good performance on predicting both therapeutic and non-therapeutic targets for the approved drugs from DrugBank and TTD. It can also reproduce 62 out of 65 new drug-target associations identified by SEA, and successfully predict both on-target and off-target interactions for 9 drugs withdrawn due to hERG toxicity. Encouraged by the results, we expect that the proposed scheme will enable large-scale target fishing, which is useful for both systematically identifying the new uses of old drugs and exploring the molecular basis of their adverse events.

## Methods

### 1. Similarity fusion for target fishing

2D fingerprint is one of the most widely used forms to represent the chemical structure in molecular similarity searching. Among various fingerprint algorithms, the extended-connectivity fingerprint (ECFP) is noteworthy due to its efficiency and the ability to capture highly specific atomic information [44]. In this study, the ECFP4 fingerprint was calculated by a component (“Molecular Fingerprints”) implemented in Pipeline Pilot 7.5 [45]. Given a query compound, its similarity score to a target is represented by a set of reference ligands is obtained by fusing the pairwise fingerprint-based molecular similarities. The similarity is measured by the Tanimoto coefficient [46,47]. For a given target *j* with *N*_*j*_ reference ligands, the following scores are calculated by different similarity fusion schemes:

(I) KNN score (KS_j_) is the average similarity of K most similar ligands of the target *j* to the query;

(II) Max score (MS_j_) is a special case of KNN when K equals to 1, which only considers the most similar ligand of the target *j* to the query;

(III) Centroid score (CS_j_) is the average similarity of N_j_ ligands of the target *j* to the query.

**Figure 7:**
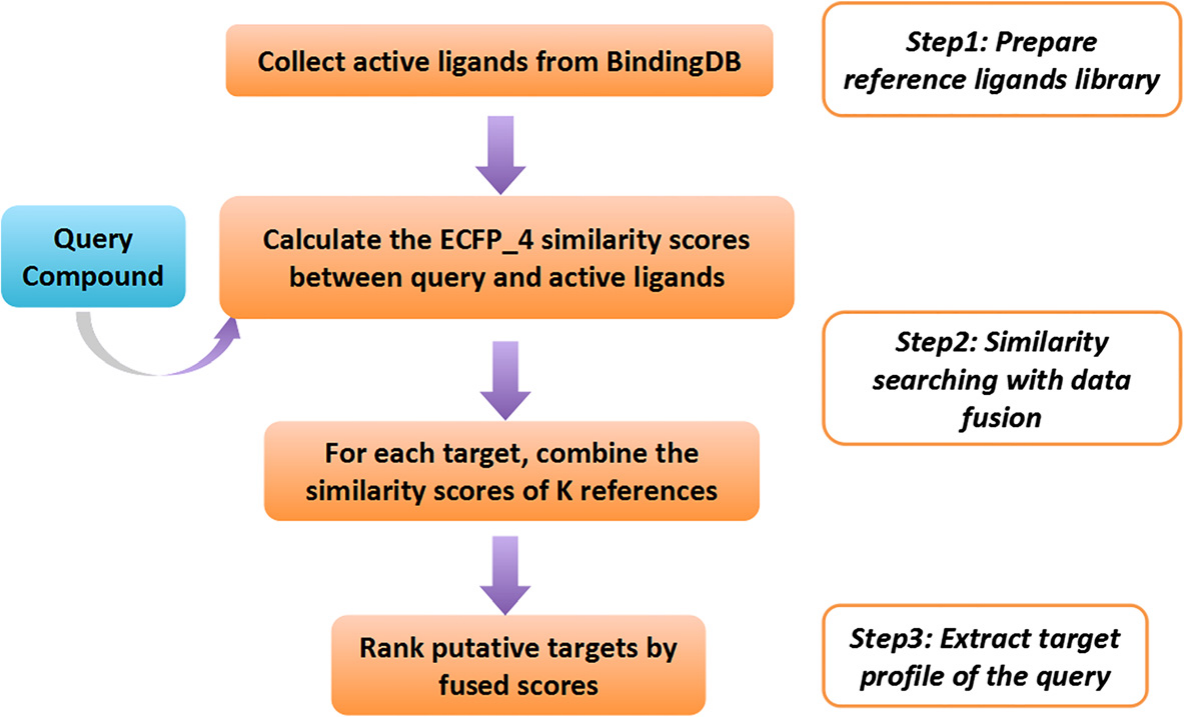
The flowchart of the ligand-based similarity-ranking scheme with data fusion.

Figure 7 outlines the target fishing workflow. The first step is to elaborately prepare a well-curated reference library that covers 533 targets represented by their active ligands as comprehensive as possible. Then, for a given query compound, 2D fingerprint based similarity searching runs through the entire sets, and the fusion scores of each target are calculated. Altogether four types of fusion scores were calculated, which are KS_j_ MS_j_ and CS_j_. Finally, for each fusion score, all the 533 targets were ranked in a descending order, and the top ranked targets were regarded as potential targets of the query. The predictive performances of different types of fusion score were compared with a 10-fold cross-validation test, in terms of the evaluation metrics defined in the next section.

### 2. Evaluation metrics

The metrics we used are defined below:

(1) $$PR_{n}=\frac{TP_{n}}{n}$$

(2) $$RE_{n}=\frac{TP_{n}}{m}$$

(3) $$F_{n}=2.\frac{PR_{n}\cdot RE_{n}}{PR_{n}+RE_{n}}$$

(4) PR'=1m∑i=1mPRi

In this study, *PR*_*n*_ (*eq.*1) means the fraction of positive predictions that are “true” (experimentally verified targets) where *TP*_*n*_ is the number of true positive prediction in the top ranked *n* targets; *RE*_*n*_ (*eq.*2) means the fraction of the “true” targets that can be recognized (predicted as positive). Both *PR*_*n*_ and *RE*_*n*_ are therefore based on an understanding and measure of a model's ability to identify true targets. *F*_*n*_ (*eq.*3) is the harmonic mean of *PR*_*n*_ and *RE*_*n*_, and a higher *F*_*n*_ score means a better performance on discriminating true targets based on an overall consideration.

The *PR’* was introduced by Amini *et al.*[36] For every correctly predicted target that appears at the *i-*th position in the top *m* ranked targets, which corresponds to the number of true targets of the ligand, the precision value at that position *PR*_*i*_ was calculated. *PR’* is given by the averaged precision values *PR*_*i*_ from the ranking places 1 to *m* (*eq.*4). According to this definition, the relevant targets that do not appear in the top *m* ranked targets receive a precision score of 0. In the end, the averaged values of the *PR*_*n*_, *RE*_*n*_, *F*_*n*_ and *PR’* for all compounds of validation datasets were reported.

## Competing interests

The authors declare that they have no competing interests.

## Authors’ contributions

Conceived and designed the experiments: MYZ and XML. Performed the experiments: XL, XY, SSL, YLW and JLP. Analyzed the data: XL, MYZ. Wrote the paper: XL, MYZ. All authors discussed the results and commented on the manuscript. All authors have given approval to the final version of the manuscript.

## Supplementary Material

Additional file 1: Table S1 The *PR*_*n*_ and *RE*_*n*_ values of 3NN and SEA for DrugBank and TTD validation sets respectively. **Table S2.** The *F*_*n*_ score of 3NN and SEA for the DrugBank and TTD sets respectively. Click here for file
