# Supplementary material for: In Silico target fishing: addressing a “Big Data” problem by ligand-based similarity rankings with data fusion
Source: J Cheminform. 2014 Jun 18;6:33. doi: 10.1186/1758-2946-6-33 (PMC4068908; doi:10.1186/1758-2946-6-33)
Supplement: Additional file 1: Table S1 — The PR n and RE n values of 3NN and SEA for DrugBank and TTD validation sets respectively. Table S2. The F n score of 3NN and SEA for the DrugBank and TTD sets respectively. [file 1758-2946-6-33-S1.pdf]

***In Silico* Target Fishing: Addressing a "Big Data" Problem by Ligand-based Similarity Rankings with Data Fusion**

Xian Liu, Yuan Xu, Shanshan Li, Yulan Wang, Jianlong Peng, Cheng Luo, Xiaomin Luo*, Mingyue Zheng*, Kaixian Chen, Hualiang Jiang

Additional file 1

Table S1. The PR_n and RE_n values of 3NN and SEA for Drugbank and TTD validation sets respectively.

	**Drugbank**				**TTD**			
	PR_n		RE_n		PR_n		RE_n	
**n**	**3NN**	**SEA**	**3NN**	**SEA**	**3NN**	**SEA**	**3NN**	**SEA**
1	0.475	0.319	0.094	0.070	0.396	0.282	0.170	0.126
2	0.405	0.253	0.138	0.097	0.312	0.228	0.237	0.179
3	0.362	0.217	0.171	0.119	0.268	0.192	0.289	0.217
4	0.327	0.178	0.193	0.125	0.231	0.157	0.317	0.230
5	0.300	0.150	0.212	0.128	0.203	0.132	0.347	0.238
6	0.279	0.128	0.230	0.129	0.179	0.113	0.366	0.246
7	0.259	0.112	0.243	0.130	0.159	0.099	0.380	0.250
8	0.242	0.099	0.255	0.131	0.145	0.088	0.392	0.251
9	0.231	0.088	0.266	0.131	0.132	0.079	0.401	0.253
10	0.221	0.080	0.276	0.132	0.123	0.071	0.412	0.254
11	0.211	0.073	0.283	0.132	0.114	0.065	0.421	0.254
12	0.201	0.068	0.292	0.132	0.107	0.060	0.429	0.254
13	0.193	0.063	0.296	0.132	0.101	0.055	0.439	0.255
14	0.186	0.058	0.302	0.133	0.096	0.052	0.445	0.255
15	0.179	0.054	0.307	0.133	0.091	0.048	0.457	0.255
16	0.173	0.051	0.314	0.133	0.088	0.045	0.465	0.255
17	0.169	0.048	0.319	0.133	0.084	0.042	0.471	0.255
18	0.164	0.045	0.324	0.133	0.080	0.040	0.477	0.255
19	0.158	0.043	0.327	0.133	0.077	0.038	0.480	0.255
20	0.153	0.041	0.330	0.133	0.074	0.036	0.483	0.256

Table S2. The F_n score of 3NN and SEA for the Drugbank and TTD sets respectively.

	Drugbank		TTD	
**n**	**3NN**	**SEA**	**3NN**	**SEA**
1	0.157	0.114	0.238	0.174
2	0.206	0.140	0.269	0.201
3	0.232	0.154	0.278	0.203
4	0.243	0.147	0.267	0.187
5	0.249	0.138	0.256	0.170
6	0.252	0.129	0.240	0.155
7	0.251	0.120	0.224	0.142
8	0.248	0.112	0.212	0.130
9	0.247	0.106	0.199	0.120
10	0.246	0.100	0.189	0.111
11	0.242	0.094	0.180	0.104
12	0.238	0.090	0.171	0.097
13	0.234	0.085	0.164	0.091
14	0.230	0.081	0.157	0.086
15	0.226	0.077	0.152	0.081
16	0.223	0.074	0.147	0.077
17	0.221	0.070	0.142	0.073
18	0.217	0.068	0.137	0.069
19	0.213	0.065	0.132	0.066
20	0.209	0.062	0.128	0.063
